# Supplementary material for: Association between oral microbiome and seven types of cancers in East Asian population: a two-sample Mendelian randomization analysis
Source: Front Mol Biosci. 2023 Nov 21;10:1327893. doi: 10.3389/fmolb.2023.1327893 (PMC10702768; doi:10.3389/fmolb.2023.1327893)
Supplement: Supplementary file 1 [file DataSheet1.ZIP › Supplementary Materials/MR plots for tongue/tongue═╝/Pancreatic cancer/╥╚╧┘░⌐_╔╕╤í╜ß╣√/pheno.3794_to_pancreatic cancer_leave_one_out.pdf]

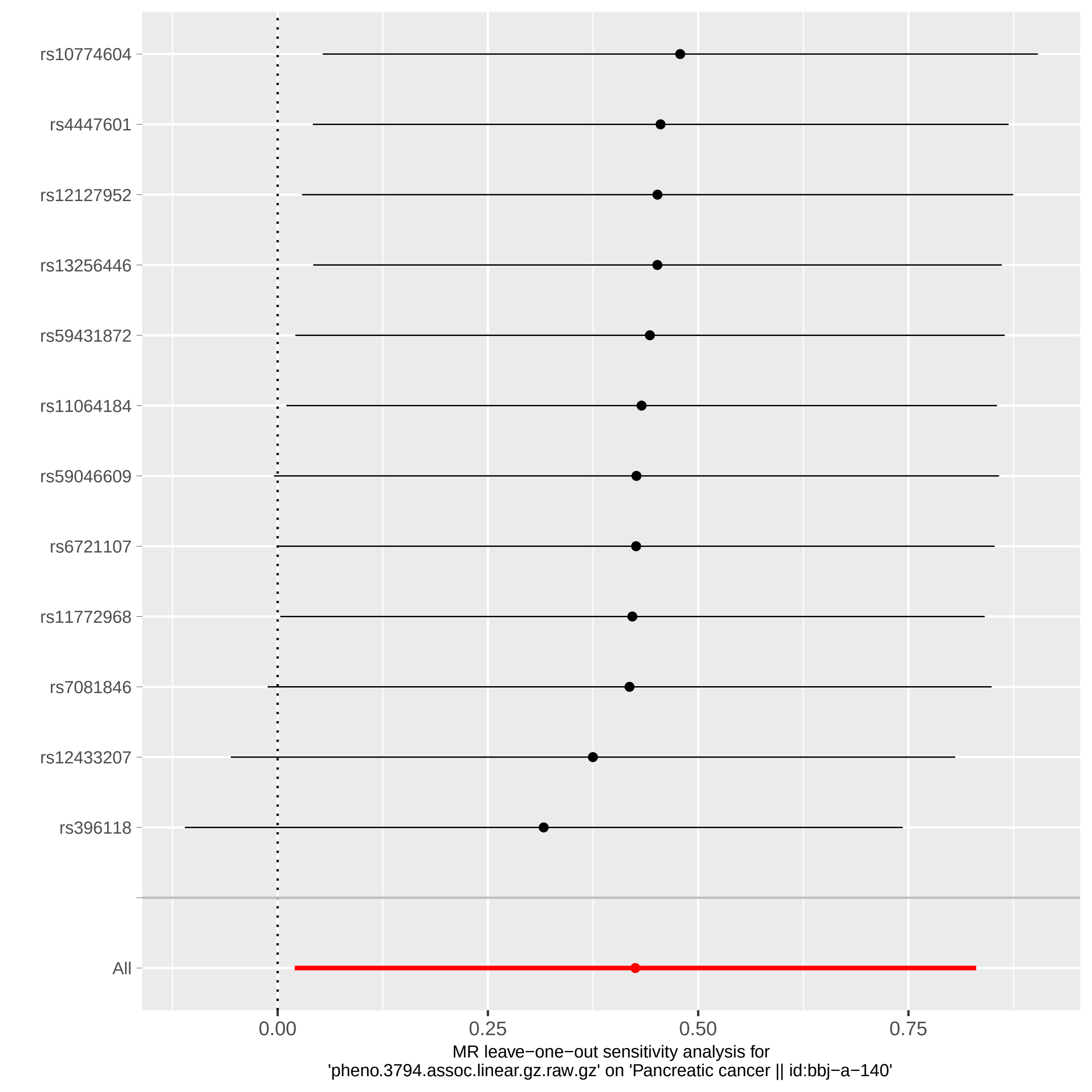
rs10774604
rs4447601
rs12127952
rs13256446
rs59431872
rs11064184
rs59046609
rs6721107
rs11772968
rs7081846
rs12433207
rs396118
All
0.00
0.25
0.50
0.75
MR leave−one−out sensitivity analysis for
'pheno.3794.assoc.linear.gz.raw.gz' on 'Pancreatic cancer || id:bbj−a−140'
